# Increased levels of neurofilament light chain in suicide attempters’ serum

**DOI:** 10.1515/tnsci-2022-0236

**Published:** 2022-08-05

**Authors:** Mahtab Ramezani, Leila Simani, Mahdiye Golestani Fard, Fatemeh Abbaszadeh, Shahin Shadnia

**Affiliations:** Department of Neurology, Loghman Hakim Hospital, Shahid Beheshti University of Medical Sciences, Tehran, Iran; Department of Pharmaceutical Sciences, College of Pharmacy, University of Kentucky, Lexington, KY 40536-0679, USA; Department of Psychiatry, School of Medicine, Loghman Hakim Hospital, Shahid Beheshti University of Medical Sciences, Tehran, Iran; Department of Neuroscience, Faculty of Advanced Technologies in Medicine, Iran University of Medical Sciences (IUMS), Tehran, Iran; Department of Clinical Toxicology, Loghman Hakim Hospital, Shahid Beheshti University of Medical Sciences, Tehran, Iran

**Keywords:** neurofilament light chain, suicidal ideation, inflammation, axonal damage

## Abstract

**Background:**

A specific biological vulnerability underlies suicidal behavior. Recent findings have suggested a possible role of inflammation and neuroaxonal injury. However, the relationship between inflammation and clinical symptoms in this disorder is still unclear. The objective of this study is applying novel blood markers of neuroaxonal integrity such as neurofilament light chain (NfL) and comparing the results with the healthy control subjects.

**Methods:**

In this cross-sectional study patients with suicide attempts were evaluated. The serum concentration of NfL on admission was measured by enzyme-linked immunosorbent assays.

**Results:**

A total of 50 patients with a suicide attempts and 35 healthy controls were included in the study. The levels of NfL in attempted suicide patients were significantly higher in comparison with healthy controls (40.52 ± 33.54 vs 13.73 ± 5.11, *P* < 0.001). A significant association between serum levels of NfL and risk factors for suicide was not found.

**Conclusion:**

These findings indicate that axonal damage may be an underlying neuropathological component of suicide attempt patients, although no correlation was observed with clinical features. This line of work could lead to new horizons in understanding the neurobiology of suicidal attempts and the development of better management strategies for these patients.

## 1 Introduction

According to the World Health Organization, over 800,000 people commit suicide every year around the world [1]. In nonfatal suicide attempts, the number may be between 10 and 20 times higher [1]. In up to 90% of suicide cases, psychiatric disorders like mood disorders, psychosis, or substance abuse are present [2]. Early detection and treatment of these disorders are the most effective ways to prevent suicide [2].

The possibility of immunological abnormalities has been shown in biomarker studies in suicidal patients [3–5]. A number of studies support the presence of inflammatory alterations in major depressive disorder (MDD) [6,7], schizophrenia [8], and bipolar disorder [9]. By supporting synaptic remodeling [10], neurogenesis, neurological circuitry, neurotransmitters, and synaptic integrity, cytokines play a main role in the development and healthy function of the brain [11].

The neurofilament light chain (NfL) is a subunit of neurofilament that is highly expressed in the axon and dendrites, where it contributes to structural stability in neurons [12]. A neuroaxonal injury due to neuroinflammatory, neurodegenerative, traumatic, or vascular damage causes NfL to be released in large quantities. This NfL enters the interstitial fluid, which communicates freely with the cerebrospinal fluid (CSF) and blood [12,13]. As reported by Disanto et al., NfL concentrations in the blood are nearly 40-fold lower than those in the CSF [13]. Nevertheless, the NfL measure in blood was proposed as a prognostic and monitoring tool for a broad range of neurological and neuropsychiatric disorders [14,15], including multiple sclerosis, Alzheimer’s, and traumatic brain injuries [12,13,16].

A growing body of evidence suggests that MDD and bipolar disorder are associated with neuroinflammation and neuroaxonal damage [17–20]. An assessment of myelinated axons found a clear reduction in myelin of the callosal splenium in patients with MDD compared to control participants [19]. The chronic stress model for depression in animals showed fewer synapses and myelinated axons [17].

There are some promising biochemical results in the area of suicidal behavior that can be used to construct an initial biological model for prediction [21]. However, studies on the association between NfL and suicide attempts are limited. Following the observation that neuroaxonal injury has been implicated in mood disorders, and that NfL has been identified as a biomarker of neuroaxonal injury in various neurological conditions, we explored the hypothesis that suicidal behavior is accompanied by an increase in NfL values, possibly suggesting its role as a biomarker in suicidality. Currently, there is no clinical predictor available, and no biomarkers have been established to aid clinicians in predicting suicidal behavior or identifying potential targets for treatment.

## 2 Methods

This analytical cross-sectional study was performed on 50 patients and 35 healthy control subjects. Cases were selected from patients admitted to the toxicology unit of Loghman-Hakim Hospital, Tehran, Iran. The comparison group consisted of healthy volunteers with no history of suicide and/or psychiatric problems (self-declared).

Patients with a confirmed diagnosis of suicide, determined by the clinical assessment and according to the Diagnostic and Statistical Manual of mental disorders fifth edition criteria for bipolar disorder and MDD, were recruited. Age, sex, pre-morbid risk factors potentially predisposing to suicide (e.g., a history of physical abuse [i.e., corporal punishment or any physical injury resulting from aggressive behavior towards the patient], sexual abuse [rape], family dysfunction [i.e., divorce, single parent, significant family disputes, etc.], academic failure [school dropout or repeated grades], history of suicide attempt, and family history of suicide) of all suicide patients were registered. Other clinical and demographic variables were extracted from the medical records. Exclusion criteria included (a) patients under 18 or over 50 years of age and (b) those with other neurological or medical conditions.


**Ethical approval**: The research related to human use has been complied with all the relevant national regulations, institutional policies, and in accordance to the tenets of the Helsinki Declaration, and has been approved by the ethics committee of the Shahid Beheshti University of Medical Sciences, Tehran, Iran (ethics committee number: IR.SBMU.RETECH.REC.1400.1022).
**Informed consent**: Informed consent has been obtained from all individuals included in this study.

## 3 Blood collection and measurement of NfL levels

Venous blood samples were taken in all the patients within 24 h of admission. Five milliliters of venous blood were collected from each participant in the study and centrifuged at 4,000 × *g* for 10 min (at 4°C). Then serum was immediately separated, and aliquots were stored at −80°C.

Serum NfL levels were measured using the commercially available enzyme-linked immunosorbent assay kit, according to the manufacturer’s instructions (ZB-13164S-H9648; ZellBio GmbH, Ulm, Germany). The serum NfL levels were expressed as ng/mL. The intra-assay and inter-assay coefficients of variation were 10 and 12%, respectively.

## 4 Statistical analysis

The analysis was performed using a statistical package for the social sciences (SPSS, version 18). Kolmogorov–Smirnov test was used to assess the normal distribution of variables. The numeric and categorical variables were expressed as mean ± s.d. frequency and percentage, respectively. The independent sample *t*-test, chi-square, and one-way ANOVA were applied to analyze the differences between variables in terms of quantitative and categorical data. In addition, linear regression analysis was performed to explore the association between NfL level with attempt suicide characteristics (i.e., duration, sexual abuse, physical abuse, etc.). A *P*-value under 0.05 was considered as statistically significant.

## 5 Results

As summarized in Table 1, the case group included 50 patients with a suicide attempt, consisting of 11 men and 39 women (mean age of 28.92 ± 11.34 years). Among 50 patients who attempted suicide, 28 (56%) persons had bipolar disorder and 22 (44%) subjects had a MDD. The disease duration before the final diagnosis was 6.56 ± 6.39 years. The comparison group was 35 volunteer healthy subjects, 10 men and 25 women with 30 ± 6.27 years on average. No significant differences were found in the main demographic variables (i.e., sex and age) between the groups. However, the groups differed significantly regarding some characteristics (e.g., education level, current employment, and dysfunctional family).

**Table1:** Characteristics and clinical features of study groups

Variables		Case group (*N* = 50)	Comparison group (*N* = 35)	*P*-value
Sex (*N*, %)				
Female		39 (78)	25 (71.4)	0.489
Male		11 (22)	10 (28.6)	
Age (mean ± s.d.^^^)		28.92 ± 11.34	30 ± 6.27	0.611
Diagnosis DSM-V (*N*, %)				
Bipolar disorder		28 (56)	NA^*^	—
MDD		22 (44)		
Disease duration (years, mean ± s.d.)		6.56 ± 6.39	NA	—
Education years (mean ± s.d.)		10.72 ± 2.38	13.17 ± 2.9	0.001
Sexual abuse (*N*, %)		13 (26)	NA	—
Physical abuse (*N*, %)		14 (28)	NA	—
Dysfunctional family (*N*, %)		25 (50)	6 (17.1)	0.001
Family history of psychiatric (*N*, %)		12 (24)	NA	—
Family history of suicide		8 (16)	NA	—
History of suicide attempt (*N*, %)		18 (36)	NA	—
Premenstrual syndrome (*N*, %)		12 (24)	8 (22.9)	0.738
Postpartum depression (*N*, %)		6 (12)	NA	—
Medication (*N*, %)		9 (18)	NA	—
Current employment (*N*, %)		12 (26.1)	20 (57.1)	0.005
Conflict (*N*, %)		27 (54)	NA	—
Smoking (*N*, %)		18 (36)	NA	—
Alcoholic (*N*, %)		14 (28)	NA	—

*Not applicable; ^ Standard deviation.

**Figure 1:**
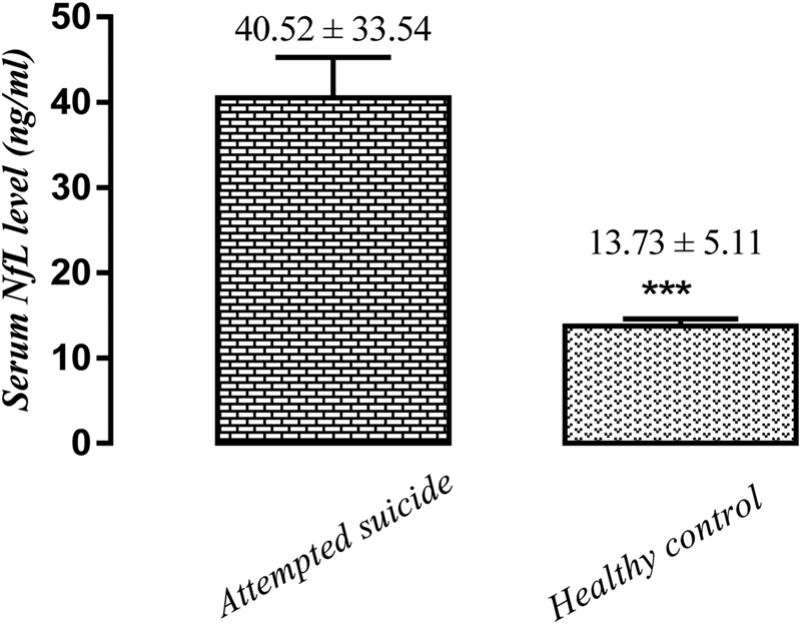
Serum NfL level in patients with attempted suicide vs control ***P < 0.001. Mean ± s.d.

Following the *t*-test, significant differences were observed in the level of NfL: patients with suicide attempts had a higher concentration compared with those in healthy control (Figure 1).

To assess whether higher levels of NfL in patients with suicide attempt is a consequence of the disease duration and pre-morbid risk factors potentially predisposing to suicide (history of physical abuse, sexual abuse, family dysfunction, academic failure, suicide attempt, and family history of suicide), the association between these variables was analyzed. There was no statistically significant association between NfL concentration and the above-mentioned variables. These results show that suicide *per se* can cause an increase in NfL compared with healthy controls.

## 6 Discussion

Early detection of individuals at high risk of self-harm can be made possible through the identification of biomarkers for suicidal behavior. A comparison of suicidal subjects with non-suicidal individuals revealed abnormalities in the monoamine system, in the hypothalamic–pituitary–adrenal (HPA) axis, lipid metabolism, inflammation, and neural plasticity. Over the past few years, studies related to the impact of adverse childhood experiences on the brain’s function have become more important, in particular genetic and epigenetic studies [22].

A study of post-mortem data from suicide victims has revealed brain changes in several cell types (neurons, glia, astrocytes, oligodendrocytes, etc.) of the cortex and subcortex [23]. Activation of the microglia may trigger increased CNS cytokine production, which can direct noradrenergic or serotonergic neurotransmission, potentially resulting in suicidal behavior [24]. According to Ducasse and colleagues’ meta-analysis, plasma levels of interleukin-2 (IL-2), IL-4, and transforming growth factor-β differ in suicidal patients and non-suicidal healthy subjects, which supports the hypothesis that altered inflammatory markers may be associated with suicidal behavior separately from mental illness [25]. There is also some evidence that plasma IL-6 levels may be associated with suicidal behaviors with violent methods, although other studies report the opposite [26].

Activation of the HPA axis can alter the level of cortisol, causing adverse effects on neurons [27]. Toll-like receptors (TLRs) are abnormally expressed by microglia, neurons, and astrocytes [28]. According to a study, suicide victims became more likely to express impaired TLR3 or TLR4 protein expression in dorsolateral prefrontal cortex (DLPFC) compared to controls. This suggests that TLR3 or TLR4 may be dysregulated in suicide victims [29]. As a result of microglia activation (e.g., DLPFC, anterior cingulate cortex [ACC], and mediodorsal thalamus), quinolinic acid production increases, and kynurenic acid production decreases, which leads to increased N-methyl-D-aspartate stimulation. Following an inflammatory state, neurotrophins, as well as the brain-derived neurotrophic factor, are diminished, which results in decrease in neurogenesis, and increased activation of the glutamatergic pathway, leading to neuronal death [30]. Activated microglia were found to be more prevalent in the white matter of the ACC and PFC of subjects who committed suicide than in subjects who died from other causes without mental disorders [31,32].

As previously discussed, mental disorders are one of the biggest risk factors for suicide [33]. An estimated 40% of those who completed suicide had previously contacted mental health services within the preceding year [34]. Neuroaxonal injury is suspected to play a pathophysiological role in MDD and bipolar disorder [35–37]. In the present study, patients with attempted suicide were diagnosed with bipolar disorder and MDD. Furthermore, our finding of increased NfL in the suicide attempt group is consistent with previous neuroimaging studies, which showed periventricular white-matter hyperintensities, and reduced anisotropy in the left orbitofrontal region and left anterior limb of the internal capsule [23]. It is important to note that increase in NfL appears to be the result of active pathological processes and not cumulative brain damage in patients with neuroinflammatory disorders [38]. NfL levels have been linked in both animal and human studies to neuroaxonal change that is macroscopically detected as white matter disruption or shrinkage of the brain [39,40]. Following suicidal behaviors, neuronal injury disintegration disrupts the blood-brain barrier and allows NfL to escape into tissues and enter the bloodstream.

According to one study, there were slight increase in NfL levels in the CSF of a subset of patients. Despite this, there was no obvious relation between NfL levels and clinical outcomes, such as manic or hypomanic episodes, suicidal attempts, or psychotic symptoms [20,41]. Similar, to previous research, we did not find significant relationships between NfL levels and several clinical variables using a linear regression model.

The causes of suicide include a diverse range of biological conditions, genetic predispositions, and environmental influences, but it is unclear if these abnormalities precede or follow suicide. To the best of our knowledge, this is the first study in suicidal patients with various underlying psychiatric disorders who showed increased NfL.

Although a single biological marker does not seem to be accurate enough to predict suicidal ideas or attempts, our results indicate that suicide attempts may induce neuroaxonal damage as demonstrated in neurodegenerative and neuropsychiatric disorders [14,15]. Research on the main molecular systems associated with suicide and their interactions with genetics, environment, and neuroplasticity is needed to improve our understanding of suicide [22].

## 7 Limitation

First, because of the small sample size of our study, the results should be interpreted with caution. Our limited financial resources restricted the use of a minimal sample size. Future studies are recommended in larger sample sizes, along with the concomitant measurements of other biomarkers. Second, psychiatric disorders and suicide are often overlapping; consequently, there is a possibility of difficulty in distinguishing between abnormalities due to suicide and those due to psychiatric diagnoses.

## 8 Conclusion

NfL in blood serum was investigated as a novel blood biomarker of neuroaxonal integrity in patients with suicide attempts. The study findings confirmed the hypothesis that patients with attempted suicide had higher levels of NfL than control subjects. Despite the lack of correlation between clinical features and levels of NfL, the role of some biological pathways in the onset of suicidal behavior could be more clearly discerned in longitudinal studies of larger samples.
